# Microbiological Evaluation of Household Drinking Water Treatment in Rural China Shows Benefits of Electric Kettles: A Cross-Sectional Study

**DOI:** 10.1371/journal.pone.0138451

**Published:** 2015-09-30

**Authors:** Alasdair Cohen, Yong Tao, Qing Luo, Gemei Zhong, Jeff Romm, John M. Colford, Isha Ray

**Affiliations:** 1 Department of Environmental Science, Policy and Management, University of California, Berkeley, California, United States of America; 2 School of Public Health, University of California, Berkeley, California, United States of America; 3 National Center for Rural Water Supply Technical Guidance, Chinese Center for Disease Control and Prevention, Beijing, China; 4 Institute of Environmental Health and Endemic Disease Control, Guangxi Zhuang Autonomous Region Center for Disease Control and Prevention, Nanning, Guangxi, China; 5 Energy and Resources Group, University of California, Berkeley, California, United States of America; Loyola University Chicago, UNITED STATES

## Abstract

**Background:**

In rural China ~607 million people drink boiled water, yet little is known about prevailing household water treatment (HWT) methods or their effectiveness. Boiling, the most common HWT method globally, is microbiologically effective, but household air pollution (HAP) from burning solid fuels causes cardiovascular and respiratory disease, and black carbon emissions exacerbate climate change. Boiled water is also easily re-contaminated. Our study was designed to identify the HWT methods used in rural China and to evaluate their effectiveness.

**Methods:**

We used a geographically stratified cross-sectional design in rural Guangxi Province to collect survey data from 450 households in the summer of 2013. Household drinking water samples were collected and assayed for Thermotolerant Coliforms (TTC), and physicochemical analyses were conducted for village drinking water sources. In the winter of 2013–2104, we surveyed 120 additional households and used remote sensors to corroborate self-reported boiling data.

**Findings:**

Our HWT prevalence estimates were: 27.1% boiling with electric kettles, 20.3% boiling with pots, 34.4% purchasing bottled water, and 18.2% drinking untreated water (for these analyses we treated bottled water as a HWT method). Households using electric kettles had the lowest concentrations of TTC (73% lower than households drinking untreated water). Multilevel mixed-effects regression analyses showed that electric kettles were associated with the largest Log_10_TTC reduction (-0.60, *p*<0.001), followed by bottled water (-0.45, *p*<0.001) and pots (-0.44, *p*<0.01). Compared to households drinking untreated water, electric kettle users also had the lowest risk of having TTC detected in their drinking water (risk ratio, RR = 0.49, 0.34–0.70, *p*<0.001), followed by bottled water users (RR = 0.70, 0.53–0.93, *p*<0.05) and households boiling with pots (RR = 0.74, 0.54–1.02, p = 0.06).

**Conclusion:**

As far as we are aware, this is the first HWT-focused study in China, and the first to quantify the comparative advantage of boiling with electric kettles over pots. Our results suggest that electric kettles could be used to rapidly expand safe drinking water access and reduce HAP exposure in rural China.

## Introduction

Globally ~1.8 billion people lack access to safe drinking water [1,2]. After decades of uneven success promoting household water treatment (HWT) using retail products such as chlorine and ceramic filters, boiling remains the most common HWT method globally [3]. Although boiling is microbiologically effective, household air pollution (HAP) from biomass and coal combustion causes cardiovascular and pulmonary disease, biomass harvesting is time-consuming, water boiled in pots is easily re-contaminated, and black carbon emissions exacerbate climate change [4–6].

Since the 1980s, China has invested heavily in rural development and drinking water infrastructure. According to Joint Monitoring Program (JMP) data, from 1990–2012, 488 million Chinese gained access to “improved” water sources (water utilities, boreholes, protected springs, etc.) [7]. However, improved sources are not necessarily microbiologically safe sources [1,2] and many smaller utilities in China do not regularly chlorinate drinking water. According to one study, as few as 10.4% of recently built water utilities in rural China consistently used disinfectants [8]. Contamination in bottled water is also a problem: 23.8% of the bottled water tested in a recent government-led investigation fell below China’s national standards for water safety [9].

There are few available national estimates of access to safe drinking water in China. According to a 2006–2007 nationwide survey led by the National Center for Rural Water Supply Technical Guidance (NCRWSTG), a specialized agency at the Chinese Center for Disease Control and Prevention (CCDC), 317 million rural Chinese lack access to microbiologically safe water [10]. More recently, the Chinese Ministry of Environmental Protection reported that 280 million (rural and urban) Chinese lack access to safe water [11].

There is a strong cultural preference for boiled water in China where 85% of the rural population (~607 million people) heats or boils its drinking water [10]. This figure includes many of the households who purchase large (19L) bottles of drinking water. In rural China, most of the population combusts biomass or coal for boiling, cooking, and heating; the resulting HAP exposure causes a number of negative health outcomes [11–14].

Despite the scale of these problems, in the water, sanitation, and hygiene (WASH) literature very little is known about HWT in China. This is in part because water quality is a politically sensitive issue in China, and in part because available data is both limited and almost all in Chinese-language sources [3]. When such sources do discuss water quality, concentrations and risk classifications are rarely provided, and instead results are reported as being over or under official standards (see S1 Text for more details). This study was born from a desire to better understand existing HWT methods and their effectiveness in rural China, with the long-term goal of expanding access to safe water while reducing HAP exposure.

## Methods

### Field sites, study design, sample size, and village selection

Given our research focus on HWT in relatively poor rural areas of China, we selected the Guangxi Zhuang Autonomous Region (Guangxi) for our study. Home to ~47 million people, Guangxi’s per capita GDP is one of the lowest among China’s provinces. Based on data from their unpublished National Rural Environmental Health Monitoring survey, the Guangxi CCDC estimated that the boiling prevalence in rural Guangxi was 68%. We used this figure for our sample size calculations to estimate the prevalence of boiling with a desired precision of ±5%. There was no prior research or data available to calculate the intracluster correlation coefficient (ICC). We used rough estimates of boiling prevalence from our pilot work to estimate an ICC of 0.01 (in order to calculate the design effect). Using a constant of 30 households per village, this yielded a required sample size of 431 households. Our final sample was 450 households (effective ICC = 0.012, design effect = 1.35 (see S2 Text)).

In light of the sensitive nature of water quality data in China, we agreed to use codes for county and village names in all publications and presentations. One benefit of this arrangement was that, because no one outside our project team would know which specific counties and villages had been selected, Guangxi county CCDC staff were comfortable providing feedback and critiques throughout our work. While not ideal, using codes for household research of this nature is not atypical in China.

We used a geographically stratified, population-weighted, multi-stage, cross-sectional design to select our study villages. After working with the Guangxi CCDC to select a relatively low-income county (County A) and a higher income county (County B), we used lists with up-to-date population data and population-based proportional sampling to randomly select study townships and then 15 study villages. The mean 2012 reported annual income for villages 1–8 in County A was RMB 4,425 (USD 702), and for villages 9–15 in County B it was RMB 6,912 (USD 1,097).

### Surveys, household sampling, and participant eligibility

For our primary survey, we used the Multidimensional Poverty Assessment Tool (MPAT) Household Survey [15]. MPAT was designed to collect a wide range of household-level data, has already been extensively tested in rural China [16,17], and is an open-source tool (all materials are available at www.ifad.org/mpat). We also developed new survey modules on water use, behaviors, attitudes [18,19], fuel use, and household ventilation; we added these to the end of the MPAT survey. Following validation of the Chinese MPAT surveys, and double-blind translation of the new survey questions, we conducted multiple rounds of piloting in a non-study county in June 2013.

After enumerator training and field practice, in August 2013, during the rainy/summer season, we surveyed 450 households in villages 1–15 (30 households per village). In December 2013 and January 2014, during the dry/winter season, we revisited four villages (two in each county) and randomly surveyed an additional 120 households. This was done to corroborate self-reported boiling data (using temperature sensors) and to address seasonal variation because we expected to find higher rates of microbial contamination during the rainy season, an assumption supported by recent research [20] (and subsequently by our summer-winter data comparisons).

Our team used local government data to prepare numbered tabs of paper corresponding to all the households in each village. Village leaders were asked to randomly select 30 tabs out of a basket, with additional households then selected to address non-response. Since this was done in public, selected households often heard of our visit and understood they had been chosen by chance (“like a lottery”) before enumerators visited their homes and read the consent statement. Households were eligible for participation if an adult (age ≥18 years) who lived in the household (>9 months/year) was available and consented to participate (<4% of households refused). Other details of the training and sampling procedures we used are explained in the MPAT User’s Guide [15].

### Water sampling, analyses, and temperature logging

Enumerators asked respondents to provide a cup of water as if the respondent were going to drink it. 500mL samples were collected aseptically and taken on ice to the county CCDC laboratories within six hours (in most cases). We did not control for chlorine residuals since we knew that, in practice, none of the local treatment plants or households used chlorine. Experienced CCDC laboratory staff used Multiple Tube Fermentation, the CCDC’s official method for the microbiological analysis of drinking water [21], to determine the Most Probable Number (MPN) of Total Bacteria (i.e., total aerobic bacteria count), Total Coliforms (TC), and Thermotolerant Coliforms (TTC) per 100mL for each sample (see S3 Text for protocol details). Following World Health Organization (WHO) guidelines, we used TTC as an indicator of fecal contamination in drinking water [22].

To assess potential physicochemical contamination, CCDC enumerator supervisors collected 5L samples from each village’s primary drinking water source(s). Samples were analyzed in the CCDC county laboratories for Chloride (Cl-), Fluoride (F-), Iron (Fe), Nitrate (NO3-), pH, Sulfate (SO4), total hardness, and turbidity (see S3 Text for details). During the winter data collection, the lead author also collected village samples and analyzed them for Aluminum (Al), Fe, NO3-, Nitrite (NO2-), Phosphate (PO4), and SO4, using a Hach CEL/850 Basic Drinking Water Laboratory (Hach, Loveland, CO).

To corroborate self-report boiling frequency and duration data, during the winter data collection we affixed Stove Use Monitoring System (SUMS) temperature loggers [23] to pots and kettles in a subsample of 47 households, recording one temperature reading (range 0–125°C at ±1°C precision) per minute over 72 hours.

### Conceptual framework for causal pathway analysis and modeling

**Fig 1:**
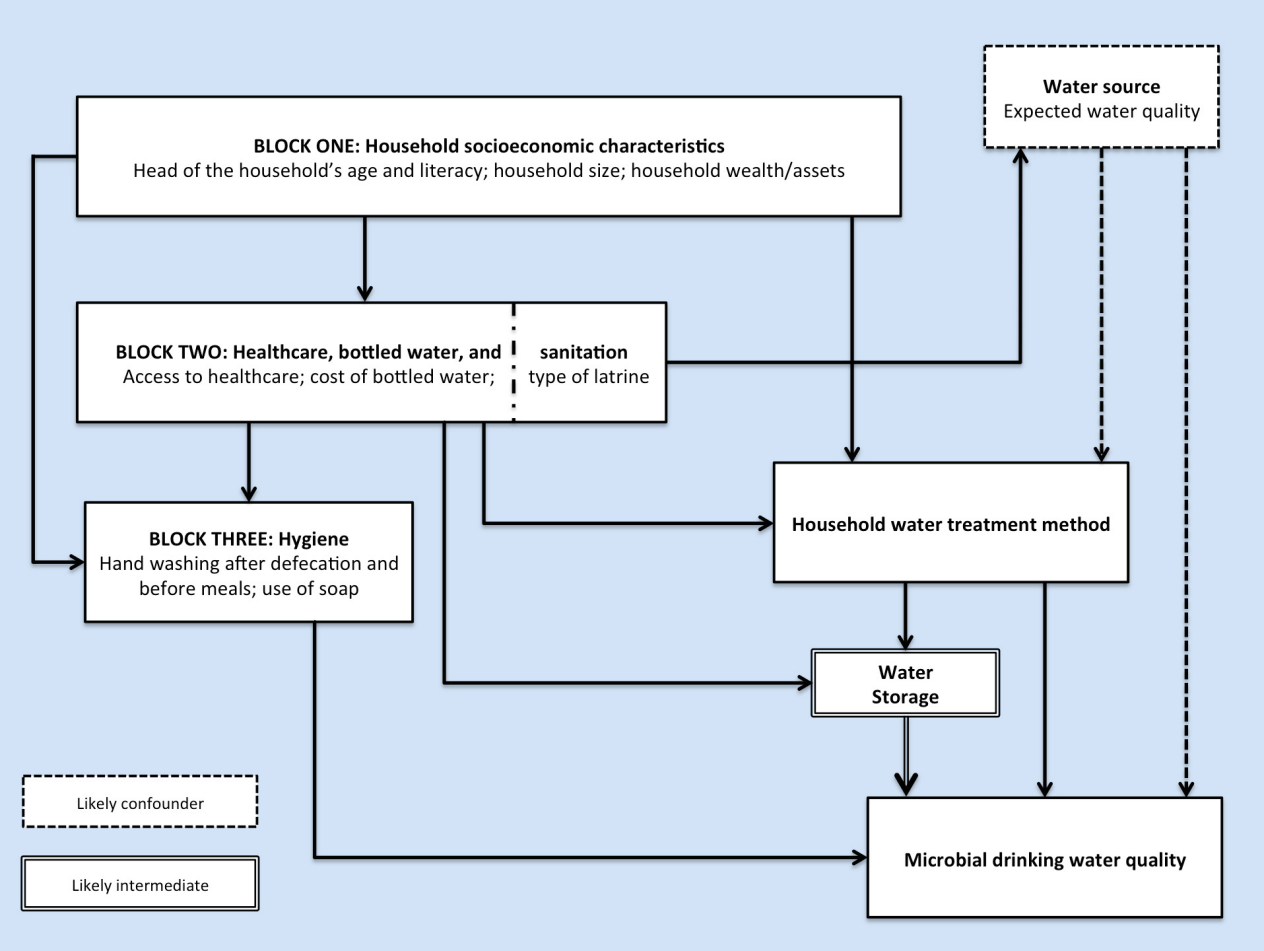
Simplified hierarchical conceptual framework of the primary factors that may impact the microbial contamination of drinking water.

Intermediate and confounding variables can mask or dampen the effect of an exposure (e.g., HWT) on an outcome (e.g., microbial water quality); as such, direct effects cannot be confidently estimated if such variables are not also analyzed in isolation [24,25]. Conceptual hierarchies help to visualize how distal determinants (usually socioeconomic variables that remain stable in the short term) may impact an outcome via proximate determinants (usually factors that may be more amenable to interventions, such as the decision to treat drinking water or not) [26,27]. Fig 1 shows the hierarchical conceptual framework we used to guide the step-wise construction of our models. The hypothesized intermediate variable “water storage” (safe = covered, unsafe = uncovered), and confounding variable “water source” (improved or unimproved), are highlighted.

### Data preparation and water quality outliers

Completed surveys were subjected to a three-stage quality control process [15] and data entry was performed by staff at the NCRWSTG. Internal data consistency was assessed and two surveys from each village were randomly selected to manually check data entry accuracy. We used a Log_10_ transformation for the TC and TTC data after assigning a value of one to all cases where TC or TTC were below the detection limit.

We identified 38 coliform-related outlier cases in the summer data (from 444 out of 450 households with TC and TTC data available). Of these outliers, 31 showed very high concentrations of TC (>2,000 MPN/100mL) but the corresponding TTC from the same samples were below the detection limit or <2 MPN/100mL. The other seven outliers had TTC to TC ratios ≥1, indicating the same or more TTC contamination versus TC (a highly unlikely outcome).

### Statistical analyses and multilevel mixed-effects modeling

For the summer data (n = 450), we applied sampling weights for population parameter estimation to balance the 240 households in County A and 210 households in County B. Missing data were ignored. We performed water quality related analyses with and without TTC outliers.

Our surveys included multiple overlapping questions related to drinking water and fuel use, allowing us to verify rates of primary (i.e., most of the time) HWT method use, and to disaggregate boiling by electric kettles and pots (i.e., metal pots or kettles, heated with wood, gas, crop residue, or coal). Initial analyses consisted of t-tests, Wilcoxon rank-sum, ANOVA, Bonferroni tests, Scheffe’s tests, and assorted bivariate analyses. Bland-Altman plots were used to compare SUMS data to self-reported winter boiling data.

Because our study households were deliberately clustered in villages, they could not be considered independent units of analysis. Therefore, we used mixed-effects multilevel regression modeling (MLM) to examine the impact of HWT (the exposure) and other variables on the dependent variable, Log_10_TTC (the outcome), while controlling for confounders and intermediates. The structure of our models can be better understood by referencing the null/unconditional variance components model (Eq 1).

$$yLog_{10}TTC_{ij}\;=\;\beta_{1}+\zeta_{j}+\epsilon_{ij}$$ (1)

Assuming,

*ϵ*_*ij*_ *~N (0*, *θ)*, with *ζ*_*j*_ *a*s the cluster-level error term

*ζ*_*j*_ *~N (0*, *ψ)* for village *j*, *j* = 1,2,3…15 (J is the total number of villages and *β* is the regression coefficient).

In Eq 1, *i* represents each individual household and *j* represents each individual village (i.e., level-one unit *i* is clustered in level-two unit *j*). MLM allows us to break down the errors into village-level residuals, denoted *ζ*, and household-level residuals within the clusters, denoted *ϵ*. The between-cluster variance is denoted *ψ*. Similar to the interpretation of residuals in ordinary least squares (OLS) regression, the household-level residual *ϵ*_*ij*_ indicates the deviation from the cluster (village) mean; this within-cluster variance is denoted *θ*. Thus, the total residual error for a household *i* in village *j* is the sum of *ζ*_*j*_ and *ϵ*_*ij*_. Assuming these two residuals are independent, the total residual variance is the sum of the variance components *ψ* and *θ*. Because we had a relatively small number of clusters (J = 15), and our data were balanced across clusters (30 households per village), we used Restricted Maximum Likelihood Estimation (REML) which provides less biased variance component estimates, as compared to Maximum Likelihood Estimation (MLE) [28].

Following the calculation of the Null model (Eq 1), we analyzed Log_10_TTC associations with HWT methods, and with potential confounder and intermediate variables combined and in isolation (Models 1–6: S1 Table). Covariates were then added in a step-wise fashion based on the block hierarchy in Fig 1 (Models 7–10: S2 Table). For all the models, we used likelihood ratio tests to verify the need for MLM (versus OLS). For each model, R^2^ was calculated by taking the difference in total variance between that model and the Null model, and dividing by the Null model’s total variance [28]. The equation for the final, fully adjusted, model is

$$\begin{array}{l} \begin{array}{l} yLog_{10}TTC_{ij}\;=\;\beta_{1}+\beta_{2}BoilElecKettle_{ij}+\beta_{3}BoilPot_{ij}+\beta_{4}BottledWater_{ij}+\\ \beta_{5}ImprovedSource_{ij}+\beta_{6}SafeStorage_{ij}+\beta_{7}HeadHHLiteracy_{ij}+ \end{array}\\ \beta_{8}HeadHHage_{ij}+\beta_{9}TVperCap_{ij}+\beta_{10}BottledWPrice_{ij}+\beta_{11}HandwashPD_{ij}+\\ \beta_{12}SoapUsed_{ij}+\beta_{13}HandwashBM_{ij}+\zeta_{j}+\epsilon_{ij} \end{array}$$ (2)

Assuming,

*ζ*_*j*_ | ***x***_*ij*_ *~ N (0*, *ψ)*

*ϵ*_*ij*_ | *ζ*_*j*_, ***x***_*ij*_ *~ N(0*, *θ)*

In Eq 2, *i* are households, *j* are clusters (villages), *ζ* are cluster-level residuals, *ϵ* are household-level residuals, *ψ* is the between-cluster variance, and *θ* is the within-cluster variance. The zero mean and variance assumptions are conditional on the model covariates, denoted by vector **x**_ij_.

We attempted to validate our models quantitatively and qualitatively. In November 2014, a groundtruthing meeting was held in Nanning (capital of Guangxi) with Guangxi CCDC staff from both counties, as well as the provincial headquarters, to discuss initial analyses and results. Feedback was used for model revisions and additional analyses. Model diagnostics and analysis of residuals were also performed. Sensitivity analysis for Model 10 was conducted using: OLS; MLE; MLE with sample weights; a more lenient use-of-soap covariate; a stricter safe water storage covariate; without safe water storage and water source; with TTC outliers; and with a number of other controls. Final modeling and analyses were performed using STATA version 13.1 (Stata Corporation, College Station, TX).

### Ethics

This study was approved by the Committee for the Protection of Human Subjects at the University of California Berkeley (protocol ID: 2012-05-4368) and by the CCDC’s Institutional Review Board (IRB) in Beijing. All participants provided informed written consent (note: Berkeley’s IRB approved the use of unsigned/verbal consent, but the CCDC’s IRB required signed consent). This manuscript was prepared using the STROBE guidelines [29].

## Results

Demographic characteristics in our sample were similar across the two counties (Table 1) and closely in line with village-level government data, indicating a representative sample (S3 Table). Our prevalence estimates for the population were that 47.5% (95% CI = 37.7–57.2) of households boiled locally sourced water for drinking, 34.4% (95% CI = 22.2–46.5) purchased bottled water, and the remaining 18.2% (95% CI = 11.3–25.0) did not treat their drinking water. Disaggregating boiling methods, we found that 20.3% (95% CI = 11.5–29.1) of all households used pots, and 27.1% (95% CI = 17.2–37.0) used electric kettles (<5% of whom used small metal kettles placed on electric burners) (Table 2 and S4 Table). While bottled water is not usually considered a HWT method, we treated it as such for these analyses, in part because at least half of the households purchasing bottled water in our sample regularly heated or boiled it before drinking (see S2 Text). After including bottled water users who likely heated or boiled their bottled water, our overall prevalence estimate for boiling was 63.13% (95% CI = 57.28–68.97); the CCDC’s 68% boiling prevalence estimate for Guangxi falls within this confidence interval.

**Table 1:** Survey, demographic, and socioeconomic characteristics by County.

	County A	County B	Total^a^
**Survey overview**			
HHs surveyed (village codes)	240 (1–8)	210 (9–15)	450 (1–15)
Survey duration in minutes: mean (±SD)	42.6 (±7.8)	38.7 (±6.7)	40.7 (±7.4)
Total population in sampled HHs (unadjusted)	1,202^a^ (1,288)	1,195^a^ (1,121)	2,397 (2,409)
Respondent gender: %male (n)	49% (116)	53% (111)	51% (227)
Respondent age: mean (95% CI)	51.27 (49.3–53.3)	51.8 (49.7–53.9)	51.53 (49.4–53.7)
**Demographic**			
Head of HH gender: %male (n)	72% (171)	96% (202)	84% (373)
Head of HH age: mean (95% CI)	51.03 (49.4–52.7)	53.95 (52.4–55.6)	52.49 (50.9–53.9)
Adults >15years in HH: mean (95% CI)	3.55 (3.4–3.7)	3.59 (3.4–3.8)	3.57 (3.3–3.8)
Children <15years in HH: mean (95% CI)	1.08 (.94–1.2)	1.06 (.89–1.2)	1.07 (.97–1.2)
Adults & children in HH: mean (95% CI)	4.63 (4.4–4.9)	4.65 (4.4–4.9)	4.64 (4.3–5)
**Socioeconomic**			
Mean annual income RMB (±SD)^b^	4,425 (±769)	6,912 (±994)	5,668 (SE = 249)
Mean annual income USD (±SD)^b^	702 (±122)	1,097 (±158)	899 (SE = 39.5)
Head of HH fully literate: %(n)	49% (117)	85.5% (178)	67.5% (295)
Housing unit’s roof is cement or concrete: %(n)	97.5% (234)	99.1% (208)	98.3% (442)
TVs/HH population: mean (95% CI)	0.29 (0.27–0.32)	0.39 (0.36–0.42)	0.34 (0.32–0.37)
Minutes to nearest health clinic: mean (95% CI)	15.5 (13.8–17.1)	6.51 (6–7.1)	11.03 (8–14.1)

HH = household | SD = standard deviation | CI = confidence interval | SE = standard error

^a^ Total means, standard errors, and confidence intervals were adjusted with sample weights

^b^ Based on government data, using the mean 2012 exchange rate: USD 1 = RMB 6.3

**Table 2:** Socioeconomic and WASH characteristics by HWT method.

	Electric Kettles	Pots	Bottled	Untreated	All HHs (n)^a^
**Demographic & socioeconomic**					
Head of HH age: mean	51.99	56.73	49.79	53.11	52.40 (446)
Total HH population: mean^b^	5.62	4.94	5.45	5.21	5.35 (447)
Head of HH fully literate: %	66.67	46.15	74.68	76.00	66.82 (440)
TVs/HH population: mean	0.34	0.33	0.33	0.40	0.34 (446)
Minutes to health clinic: mean	12.39	15.63	9.09	8.84	11.31 (440)
**WASH-related**					
Improved water source: %	55.74	57.14	39.74	39.19	47.63 (443)
Store water safely: %	91.43	86.75	81.58	91.67	86.89 (412)
Water tap in/near HH (any source): %	98.35	94.38	98.73	97.33	97.5 (442)
**Sanitation & hygiene**					
Have improved latrine: %	85.25	82.80	93.59	74.67	85.87 (446)
Wash hands before meals: %	86.89	78.49	85.99	82.67	84.12 (447)
Wash hands after defecation: %	54.10	40.86	64.33	56.00	55.26 (447)
Report soap use: %	45.19	50.00	42.61	31.88	44.03 (377)
Soap, that is likely used, observed: %	42.62	47.83	47.10	25.33	42.34 (444)

HH = household

^a^ Total n excludes missing data (with no missing data n = 450) | Totals were not adjusted with sample weights

^b^ Total household population includes adults living/working outside the home >9 months/year

Nearly all households (97.5%) reported having a piped source of water in their home or courtyard (mostly from utilities, wells, rainwater harvesting cisterns, or boreholes) and 99.8% of households had access to electricity. Reported two-week diarrhea prevalence (3.8%, n = 17) was similar to that in other middle-income countries.

**Fig 2:**
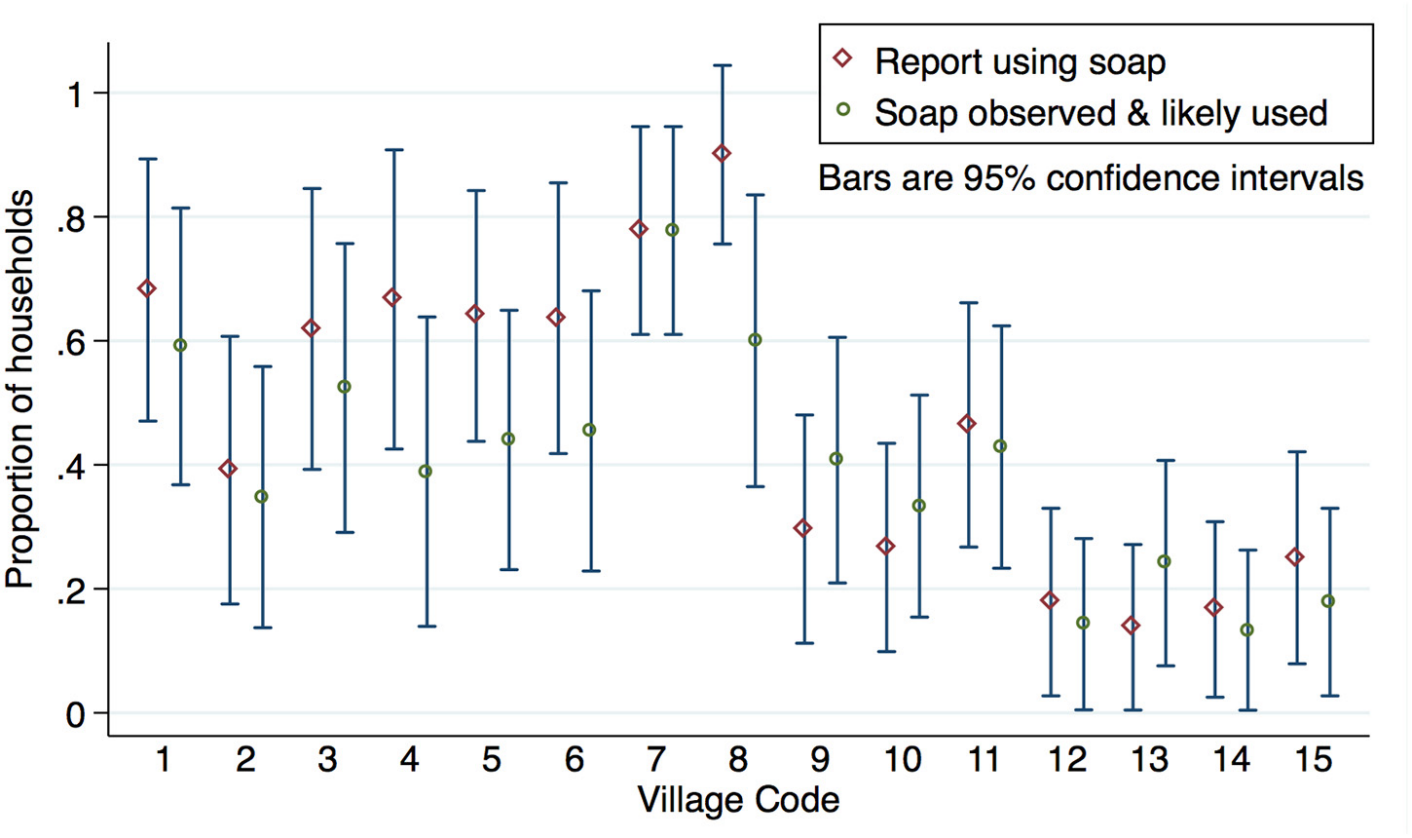
Reported and observed soap use: Proportions by village.

Courtesy bias can be a problem in self-reported WASH surveys. For example, in a study focused on hygiene and social desirability, 97% reported using soap, though it was observed in only 68% of households [30]. In our study, rates of reported and observed soap use were very similar overall (Table 2) and in most villages (Fig 2); this suggests that courtesy bias was not a significant problem. Similarly, winter self-report boiling duration and frequency data were corroborated by the SUMS data.

**Fig 3:**
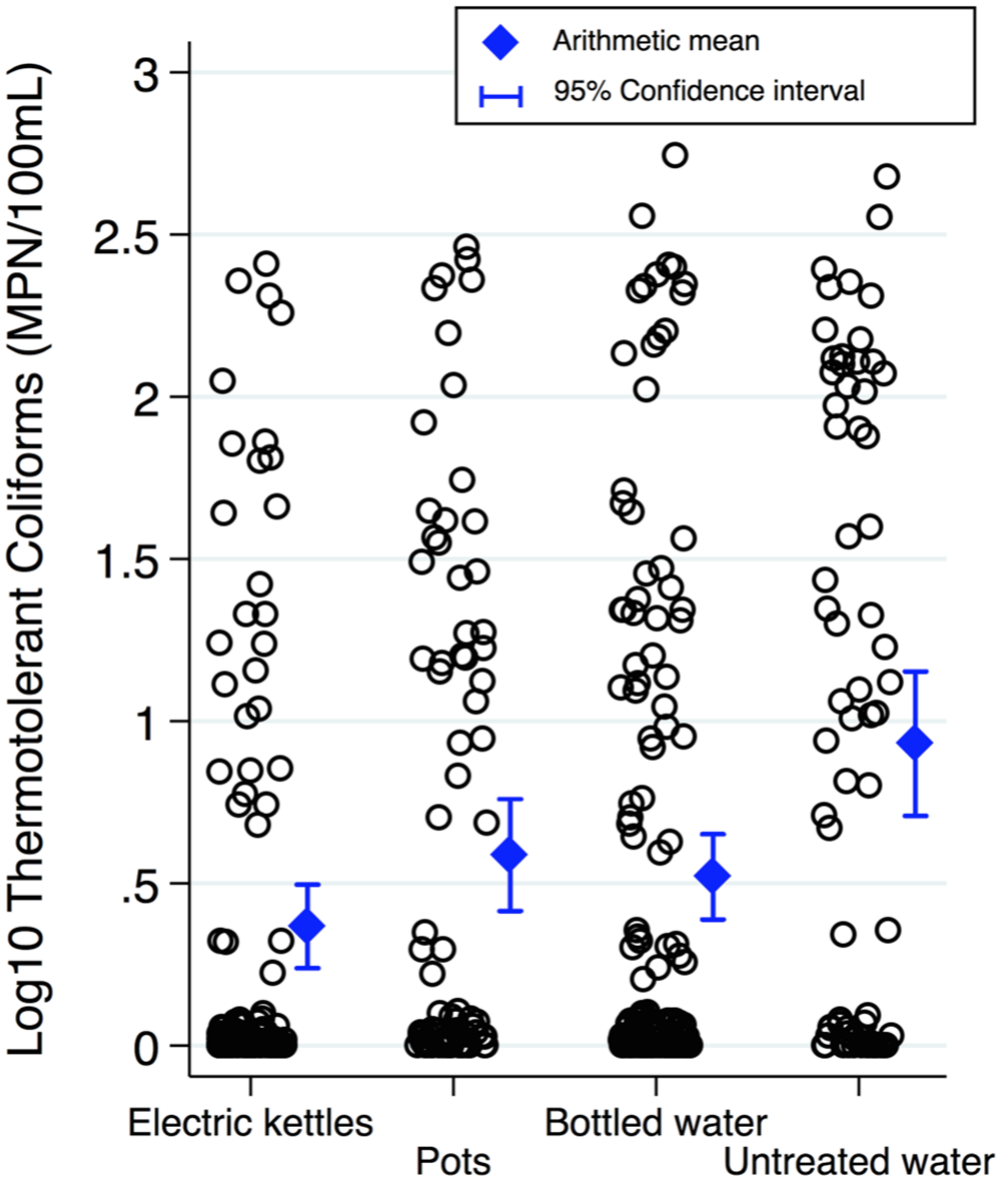
Log_10_ Thermotolerant Coliform data by HWT method. A jitter of five was used to better display observation frequencies. Data exclude 38 TTC outlier cases. Drinking water samples from households using electric kettles were associated with the lowest mean Log_10_TTC concentrations. Scheffe’s multiple-comparison test showed mean Log_10_TTC for kettles and bottled water were both statistically significantly different than untreated (p<0.001 and p<0.01, respectively); Bonferroni test showed kettles, pots, and bottled water were significantly different than untreated (p<0.001, p<0.05, and p<0.01, respectively).

For the 15 villages sampled, the mean pH of the primary drinking water source was 7.78 (SD = 0.20), turbidity was <1 for all villages, and mean total hardness was 177.6 mg/L (SD = 48.57). Aside from SO4 concentrations in two villages, none of the physicochemical variables exceeded CCDC standards (see S3 Text for additional results). TTC were detected in 38.7% of households. Across HWT methods, TTC concentrations and the proportion of households with TTC detected were both lowest for households boiling with electric kettles (see Table 3 and Fig 3). TC were detected in 93.7% of households and TC median values were lowest in the electric kettle group (see S12 Table for more details).

**Table 3:** Thermotolerant Coliform concentrations by HWT method.

Household Water Treatment Method	Sample % (n)	TTC detected: % per method (n)	Geometric mean TTC MPN/100mL	
			Mean (95% CI)	% lower than untreated
Boil: Electric kettle	27.0% (109)	28.4% (31)	2.33 (1.7–3.1)	73%
Boil: Pot	20.8% (84)	42.9% (36)	3.86 (2.6–5.7)	55%
Bottled water	34.5% (139)	40.3% (56)	3.31 (2.4–4.5)	61%
Untreated water	17.6% (71)	57.8% (41)	8.52 (5.1–14.2)	*reference*

HH = household | MPN = Most Probable Number | CI = confidence interval; Data exclude 38 TTC outliers and proportions were not adjusted with sample weights. Outlier inclusion yielded lower geometric mean TTC estimates for all three HWT methods and a higher estimate for the untreated group. Geometric means were calculated using all observations.

Table 4 displays Log_10_TTC coefficients for HWT methods adjusted only for clustering (i.e., Model Two) compared to the final, fully adjusted, model (i.e., Model Ten; see Eq 2). After controlling for the influence of likely confounders and intermediates, as well as the impact of clustering and other covariates often associated with microbial water quality, boiling with electric kettles was associated with the largest Log_10_TTC reduction (-0.60, *p*<0.001), followed by bottled water (-0.45, *p*<0.001) and boiling with pots (-0.44, *p*<0.01).

**Table 4:** Log_10_ Thermotolerant Coliform coefficients from select models.

	*Unadjusted Model*	*Final Model*
**Fixed part**		
Boil with electric kettle (vs. no)	-0.57 (0.12)***	-0.60 (0.13)***
Boil with pot (vs. no)	-0.38 (0.13)**	-0.44 (0.14)**
Drink bottled water (vs. no)	-0.45 (0.12)***	-0.45 (0.13)***
“Improved” water source (vs. no)		-0.04 (0.10)
Safe water storage (vs. no)		-0.05 (0.12)
HH head is literate (vs. no)		-0.17 (0.10)
HH head’s age (10 year steps)		0.04 (0.03)
TVs by HH population		-0.34 (0.19)
Bottled water price by village		0.72 (0.72)
Wash post defecation (vs. no)		0.07 (0.09)
Soap likely used (vs. no)		-0.06 (0.09)
Wash before meals (vs. no)		-0.20 (0.13)
Intercept	0.96 (0.10)***	0.92 (0.40)*
**Random part**		
Between-level √*ψ*	0.13 (0.06)	0.17 (0.06)
Within-level √*θ*	0.78 (0.03)	0.76 (0.03)
**Model comparison**		
Log-likelihood	-479.6	-428.1
R^2^	0.043	0.081

HH = household

* p<0.05;

** p<0.01;

*** p<0.001

Values are Log_10_TTC β coefficients with standard errors (SE) in parentheses. √*ψ* and √*θ* are the between-cluster and within-cluster standard deviation, with SE in parentheses. As model fit improves, variance and log-likelihood decrease. R^2^ indicates the linearity between covariates and Log_10_TTC, not an overall goodness of fit. The large bottled water price SE is because village means were used for all households in a village. “Improved” water source classifications were based on JMP definitions.

Model diagnostics and analysis of level-1 (household) and level-2 (village) residuals did not reveal any outlier observations. HWT effect sizes and significance levels were stable across sensitivity analysis models, and TTC outlier inclusion consistently yielded larger effects (see S5–S8 Tables). After using MLM to control for potential confounders and intermediaries (which were not significantly associated with Log_10_TTC), as well as the impact of clustering and other covariates, we found that boiling with electric kettles was consistently associated with the largest Log_10_TTC reductions. Geometric mean TTC was 73% lower for households boiling with electric kettles as compared to households drinking untreated water (Table 3).

**Fig 4:**
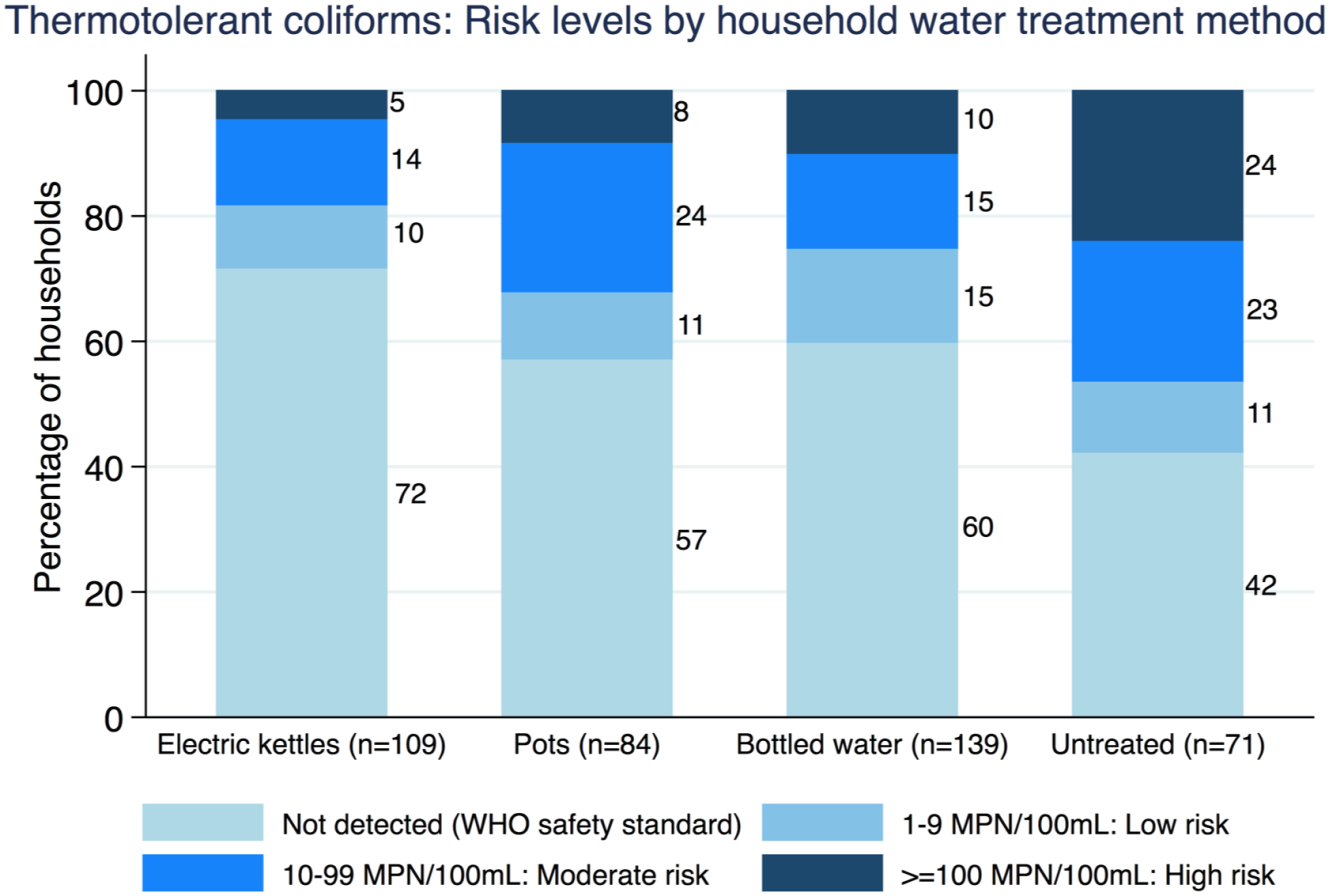
Basic risk classification of Thermotolerant Coliforms by HWT method. Each stacked bar displays TTC concentrations divided into categories based on likely health risk, and the percentage of households in each risk category by HWT method. The WHO’s standard for the microbiological safety of water using TTC as an indicator of fecal contamination is no detectable TTC/100mL [22]. The CCDC’s risk classification also considers TTC samples that are below the detection limit as microbiologically safe [21]. At counts of 1–9 MPN/100mL, if sanitary conditions are decent, drinking water is usually low risk for most people, except young children, the elderly, and the immunocompromised. Data exclude 38 TTC outlier cases (outlier inclusion yielded slightly larger proportions of households in the high risk category).

Compared to households drinking untreated water, electric kettle users had the lowest risk of having TTC detected in their drinking water (RR = 0.49, 0.34–0.70, *p*<0.001), followed by bottled water users and households boiling with pots (Table 5). Although our study was not powered to detect differences in diarrhea, the pattern of risk ratios for reported diarrhea by HWT method was consistent with the TTC risk ratios (Table 5). TTC concentration risk classifications are shown across HWT methods in Fig 4.

**Table 5:** Risk ratios for TTC and diarrhea by HWT method.

Household Water Treatment Method	TTC detected: Excluding TTC outliers		TTC detected: All data		Diarrhea reported	
	Risk Ratio (95% CI)	p-value	Risk Ratio (95% CI)	p-value	Risk Ratio (95% CI)	p-value
Untreated water	1*	n/a	1*	n/a	1*	n/a
Boil: Electric kettle	0.49 (0.34–0.70)	0.0001	0.44 (0.31–0.63)	0.0000	0.61 (0.16–2.39)	0.4781
Boil: Pot	0.74 (0.54–1.02)	0.0647	0.69 (0.51–0.93)	0.0162	0.40 (0.08–2.14)	0.2691
Bottled water	0.70 (0.53–0.93)	0.0164	0.61 (0.46–0.80)	0.0007	0.85 (0.26–2.82)	0.7936

*Reference for unadjusted risk ratios (No TTC detected = 0 | No diarrhea reported = 0)

## Discussion

Our findings in rural China are in line with the post-boiling TTC reductions observed in rural Guatemala, India, Peru, and Vietnam, as well as post-boiling *Escherichia Coli* reductions observed in Cambodia [31–35]. Other than HWT, no other covariates were significantly associated with Log_10_TTC (except for adult literacy in a few models: S2 Table). That factors such as safe water storage and latrine type were not significantly associated with water quality is likely due to the particularities of rural China, the cultural preference for boiled water, excellent access to electricity and water, and decent levels of sanitation and hygiene.

As would be expected, then, Log_10_TTC means derived from Model 10 were similar to unadjusted means (e.g., 0.35 (0.17–0.53) and 0.37 (0.24–0.49) MPN/100mL, respectively, for electric kettles). While there was no significant association between improved and unimproved drinking water sources and TTC concentrations overall, households in the untreated group using water from improved sources had significantly lower mean Log_10_TTC than households using unimproved sources (0.57 vs. 1.17 MPN/100mL; two-sided t-test with unequal variances, *p*<0.01). This finding further supports the observed overall effectiveness of HWT for microbial contaminant reduction in our study population. That said, of the HWT users, 25% reported “sometimes” or “often” drinking untreated water, highlighting the phenomenon of inconsistent HWT use [36,37].

Compared to electric kettle users, households drinking untreated water were twice as likely to have TTC detected (RR = 2.03, 1.42–2.90, *p*<0.001) and pot users were 1.5 times as likely (RR = 1.51, 1.02–2.22, *p*<0.05) (S9 Table). Respondents drinking untreated water were also more likely to report diarrhea than electric kettle users, though the association was not significant (RR = 1.63, 0.42–6.31, *p* = 0.478) (S10 Table).

There could be several reasons for the significantly lower levels of TTC in drinking water from households boiling with electric kettles. Though many pathogens are inactivated at temperatures below boiling [38], electric kettles bring water to a rolling boil for full inactivation. In addition, the kettle’s built-in lid must be closed for operation, reducing the potential for post-boiling secondary contamination [6]. Households using electric kettles also reported boiling more frequently (2.48x/day, n = 93, 95% CI = 2.10–2.87) than households boiling with pots (1.39x/day, n = 115, 95% CI = 1.20–1.57) (two-sided t-test with unequal variances, *p*<0.001) suggesting that households using electric kettles may boil smaller quantities more frequently. Among households using electric kettles, shorter boiling durations were significantly associated with lower TTC concentrations (p<0.05 with a Kruskal Wallis test, and p<0.01 for a trend test of odds ratios). This was the case even after controlling for household size and other covariates, whereas no such relationship was observed for households boiling with pots (see S4 Text). Luby et al. [39] also found that shorter boiling durations were associated with lower coliform counts. Taken together, these data suggest that, compared to pots, electric kettles provide consistent and full pathogen inactivation and limited opportunity for secondary contamination.

Our findings also highlight the presence of microbial contamination in bottled water. We found no association between price and the microbial quality of bottled water. Our survey data and qualitative feedback from the 2014 groundtruthing meeting suggest that a significant proportion of rural households purchase bottled water for its perceived convenience, rather than its perceived safety. In the population we sampled, an electric kettle of mid-range quality would cost the equivalent of seven 19L bottles of water, or 1% of mean annual reported income (RMB 55, USD 8.8). If chemical contaminant concentrations were the same or higher in bottled water as compared to local water sources, then a one-time purchase of an electric kettle would provide safer drinking water and significant long-term savings compared to bottled water.

Our study had some limitations. In deference to CCDC officials and their Institutional Review Board, some of the MPAT survey questions (and the entire MPAT Village Survey) were censored and we did not test for all industrial and agricultural contaminant indicators of interest. Due to logistical constraints related to the rainy season we replaced three of the originally (and randomly) selected villages in County A using socio-demographic matching which may have introduced some selection bias (S11 Table). In some cases water samples arrived at county laboratories after the proscribed six hours, which may partially explain the 38 coliform outlier cases. However, the inclusion of TTC outliers in our analyses resulted in larger risk ratio estimates (except for bottled water, see S9 Table) and larger Log_10_TTC reduction estimates in our models (see S8 Table). In future research, we plan to collect more data in order to more precisely disaggregate bottled water users based on their bottled water heating and boiling behaviors. Finally, with regard to HAP from boiling, cooking, and heating, it remains unclear whether removing HAP from boiling alone would result in improved health outcomes. This, too, is something we wish to investigate in future research.

## Conclusions

As far as we are aware, this is the first HWT-focused study in China. Our findings indicate that promoting electric kettles, and/or providing free or subsidized kettles to low-income households, could rapidly expand reliable access to microbiologically safe drinking water in rural China. Considering that new HWT methods face numerous adoption barriers [40–42], promoting an easier, cheaper, and faster way to practice a widely used existing HWT method could result in high uptake and sustained use. Given the scale of China’s rural population and the high baseline rate of boiling with pots and solid fuels, resulting reductions of HAP exposure and black carbon emissions could be substantial. Looking beyond China, our conclusions are pertinent to other low- and middle-income countries that are seeking safe water access through viable and scalable HWT approaches.

## Supporting Information

S1 Text Limited drinking water data for China. (DOCX)

S2 Text Intracluster Correlation Coefficient calculation. (DOCX)

S3 Text Protocols, equipment, and physicochemical results summary for water sample analyses. (DOCX)

S4 Text Details of boiling duration and TTC analyses. (DOCX)

S1 Table Log_10_TTC coefficients for Models Null-6. (DOCX)

S2 Table Log_10_TTC coefficients for Models 7–10. (DOCX)

S3 Table Sample and government data comparisons. (DOCX)

S4 Table Primary HWT method proportions: Sample and population estimates. (DOCX)

S5 Table Sensitivity analysis: HWT coefficients part I. (DOCX)

S6 Table Sensitivity analysis: HWT coefficients part II. (DOCX)

S7 Table Sensitivity analysis: HWT coefficients part III. (DOCX)

S8 Table Sensitivity analysis: Null Model and Model 10 with TTC outliers included. (DOCX)

S9 Table Risk ratios for TTC by HWT method: Electric kettles as reference (and outliers). (DOCX)

S10 Table Risk ratios for diarrhea by HWT method: Electric kettles as reference. (DOCX)

S11 Table Comparison of County A replacement and randomly selected villages. (DOCX)

S12 Table Total Coliform summary statistics by HWT method. (DOCX)
