# Supplementary material for: Microbiological Evaluation of Household Drinking Water Treatment in Rural China Shows Benefits of Electric Kettles: A Cross-Sectional Study
Source: PLoS One. 2015 Sep 30;10(9):e0138451. doi: 10.1371/journal.pone.0138451 (PMC4589372; doi:10.1371/journal.pone.0138451)
Supplement: S1 Text — (DOCX) [file pone.0138451.s001.docx]

**S1 Text: Limited drinking water data for China**

A clear example of the relative lack of drinking water quality data, and of the Chinese/English language-barrier, surfaced in response to Rosa and Clasen’s [1] estimate that ~1.1 billion people use HWT worldwide. They acknowledged their review did not include China because they were unable to find any data. Yang, Wright, and Gundry replied [2] that, according to a Chinese-language paper [3], more than 600 million Chinese use HWT, bringing the global estimate closer to 1.8 billion. The data they cited came from a 2006-2007 CCDC-led national survey, which was managed by Yong Tao [4], one of our paper’s authors.

With regard to present rates of safe drinking water access, the few available national estimates vary. The most accurate (though somewhat outdated) published estimate is from the CCDC-led national survey, suggesting that ~317 million rural Chinese lack access to safe water [3,4]. More recently, the Joint Monitoring Program of the WHO/UNICEF reported that 112 million Chinese still use “unimproved” sources [5]; however, the Chinese Ministry of Environmental Protection estimates that 280 million Chinese still lack safe drinking water [6].

When consulting Chinese-language reports and journal papers on rural drinking water (as we have done), one finds that specific water quality data is rarely provided, and most only report microbial contaminant concentrations as being “above” or “below” government standards. Bain et. al.’s recent [7] meta-analysis of drinking water quality and fecal contamination illustrates this point. They identified nine suitable studies from China; and while all of these studies used Thermotolerant Coliforms (TTC) as an indicator of fecal contamination and water quality, none provided arithmetic or geometric means, or microbial risk classification data (see the “water quality database” in their Supplemental Material; database ID numbers of the studies are: 265, 272, 273, 381, 424, 540, 543, 552, 553, 554, and 554). This is typical of most Chinese water quality studies.

1. Rosa G, Clasen T (2010) Estimating the scope of household water treatment in low- and medium-income countries. The American Journal of Tropical Medicine and Hygiene 82: 289-300.

2. Yang H, Wright J, Gundry SW (2012) Household water treatment in China. The American Journal of Tropical Medicine and Hygiene 86: 554-555.

3. Zhang R, Li H, Wu X, Fan F, Sun B, et al. (2009) 我国农村饮用水水质现状 [Current situation of Chinese rural drinking water]. 环境与健康杂志 [Chinese Journal of Environmental Health] 26: 3-5.

4. Tao Y (2009) 中国农村饮用水与环境卫生现状调查 [China Rural Drinking Water and Environmental Health Survey]. 环境与健康杂志 [Chinese Journal of Environmental Health] 26: 1-2.

5. WHO/UNICEF (2014) Progress on drinking water and sanitation: 2014 update. Geneva, Switzerland: World Health Organization.

6. MoEP (2013) 中国人群环境暴露行为模式研究报告 （成人卷） [Report of Environment Exposure Related Activity Patterns Research of Chinese Population (Adult pop.)]. Beijing: 中华人民共和国环境保护部 [People's Republic of China Ministry of Environmental Protection].

7. Bain R, Cronk R, Wright J, Yang H, Slaymaker T, et al. (2014) Fecal contamination of drinking-water in low- and middle-income countries: A systematic review and meta-analysis. PLoS Medicine 11: e1001644.
