# Supplementary material for: Microbiological Evaluation of Household Drinking Water Treatment in Rural China Shows Benefits of Electric Kettles: A Cross-Sectional Study
Source: PLoS One. 2015 Sep 30;10(9):e0138451. doi: 10.1371/journal.pone.0138451 (PMC4589372; doi:10.1371/journal.pone.0138451)
Supplement: S2 Text — (DOCX) [file pone.0138451.s002.docx]

## S2 Text. Intracluster Correlation Coefficient calculation.

To calculate the boiling ICC we created a new variable based on the observation that most of the ~35% of households who drink bottled water usually heat or boil the bottled water before drinking it (using the built in heating-element in the bottle water stands or by transferring the bottled water to a pot or kettle and then heating it). Based on other survey questions, we estimated conservatively that at least 72 of the 157 households drinking bottled water also heated or boiled that water. Those households using bottled water and identified as likely boilers had lower levels of TTC than households not identified as likely bottled-water-boilers (Log_10_TTC 0.45 MPN/100mL compared to 0.57 MPN/100mL). This lends some additional support to our estimation, though the difference was not statistically significant (perhaps due to the relatively small *n* for this subgroup analysis [one-way t-test, p=.182]).

Using this new variable and controlling for clustering, we estimated that at least 63.13% (confidence interval = 57.28-68.97%) of households in our sample regularly heat or boil their drinking water. The Guangxi CCDC estimate of 68% is therefore within our confidence interval for estimated boiling prevalence. Using this new variable, we estimated that the actual ICC was 0.059, meaning that our sample size was likely sufficient for detecting a ±7% difference in expected versus actual boiling (±6% without clustering, at power=0.8, α=0.043).
