# Supplementary material for: Microbiological Evaluation of Household Drinking Water Treatment in Rural China Shows Benefits of Electric Kettles: A Cross-Sectional Study
Source: PLoS One. 2015 Sep 30;10(9):e0138451. doi: 10.1371/journal.pone.0138451 (PMC4589372; doi:10.1371/journal.pone.0138451)
Supplement: S3 Text — (DOCX) [file pone.0138451.s003.docx]

## S3 Text. Protocols, equipment, and physicochemical results summary for water sample analyses.

**Multiple Tube Fermentation protocols**

As per the CCDC’s national standards and protocols for water quality analyses [1], a nutrient agar medium was used for the Total Bacteria (aerobic bacteria) count, incubated at 37°C for 24 hours. For the Total Coliform analysis, after a preliminary fermentation test using a lactose peptone broth at 37°C for 24 hours, plate isolation was used with an Eosin Methylene Blue (EMB) agar medium at 37°C for 24 hours, followed by a secondary fermentation test using lactose peptone broth at 37°C for 24 hours. For Thermotolerant Coliform analysis, an EC medium and EMB agar medium were used, incubated at 37°C for 48 hours.

**Equipment used by the CCDC for physicochemical analyses**

For the physicochemical analyses of village water samples, the CCDC laboratories used chromatography and spectrophotometry with a Dionex ICS-5000 Chromatograph, a Hach DR-2800 Spectrophotometer, and a NexION 300D ICP Mass Spectrometer. An AFS-3100 hydride-atomic fluorescence photometer was also used and pH was measured using a pHS-25C precision acidity meter.

**Physicochemical data results**

Mean F- was 0.2 mg/L (SD=0), mean NO3- was 1.95 mg/L (SD=1.87), mean Cl- was 9.27 mg/L (SD=5.36), mean Fe was 0.167 mg/L (SD=0.13), and the mean SO4 concentration was 10.18 (SD=15.59). At the village level, aside for SO4 in villages 12 and 13, no other variables exceeded CCDC standards. For the winter sampling, no variable exceeded CCDC standards (and the CCDC’s measurements were very similar to the lead author’s, except for SO4 concentrations in village six: 30mg/L versus the CCDC’s 7.3 mg/L).

1. MoH (2006) 生活饮用水标准检验方法: 总则 [Standard examination methods for drinking water: General principles]. Beijing: 中华人民共和国卫生部 [People's Republic of China, Ministry of Health].
