# Supplementary material for: Microbiological Evaluation of Household Drinking Water Treatment in Rural China Shows Benefits of Electric Kettles: A Cross-Sectional Study
Source: PLoS One. 2015 Sep 30;10(9):e0138451. doi: 10.1371/journal.pone.0138451 (PMC4589372; doi:10.1371/journal.pone.0138451)
Supplement: S4 Text — (DOCX) [file pone.0138451.s004.docx]

## S4 Text. Details of boiling duration and TTC analyses.

| **Boiling duration** | **Log_10_TTC arithmetic mean** | **TTC geometric mean** |
| --- | --- | --- |
| 2-4 minutes | 0.2359747 | 1.722 |
| 5-9 minutes | 0.3393368 | 2.184 |
| 10-14 minutes | 0.4018122 | 2.522 |
| 15+ minutes | 0.8144976 | 6.524 |

We used MLM to analyze the association shown in the table above. After controlling for household size, we found that for each additional minute of boiling with electric kettles there was a 0.027 MPN/100mL increase in Log_10_TTC (SE=0.012, *p*=0.03). When we controlled for all the covariates in Model Ten, the effect size and direction remained essentially the same (0.029 MPN/100mL, SE=0.014, *p*=0.036). However, when we conducted the same analysis for boiling durations among users boiling with pots, we found no such relationship (-0.001 MPN/100mL, SE=0.012, *p*=0.932).
