# Supplementary material for: Microbiological Evaluation of Household Drinking Water Treatment in Rural China Shows Benefits of Electric Kettles: A Cross-Sectional Study
Source: PLoS One. 2015 Sep 30;10(9):e0138451. doi: 10.1371/journal.pone.0138451 (PMC4589372; doi:10.1371/journal.pone.0138451)
Supplement: S1 Table — (DOCX) [file pone.0138451.s005.docx]

Table S1. Log_10_TTC coefficients for Models Null-6.

|  | ***Model Number*** | | | | | | |
| --- | --- | --- | --- | --- | --- | --- | --- |
|  | ***Null*** | ***1*** | ***2*** | ***3*** | ***4*** | ***5*** | ***6*** |
| **Fixed Part** | | | | | | | |
| Treat drinking water [vs. no] |  | -.48(.11)  *** |  |  |  |  |  |
| Boil electric kettle [vs. no] |  |  | -.57(.12)  *** |  |  | -.58(.13)  *** |  |
| Boil pot [vs. no] |  |  | -.38(.13)  ** |  |  | -.36(.14)  ** |  |
| Drink bottled water [vs. no] |  |  | -.45(.12)  *** |  |  | -.42(.12)  ** |  |
| Improved water source [vs. no] |  |  |  | -.08(.09) |  |  | -.08(.09) |
| Safe water storage [vs. no] |  |  |  |  | -.08(.12) | -.09(.12) | -.05(.12) |
| Intercept | .57(.05)  *** | .96(.10)  *** | .96(.10)  *** | .60(.07)  *** | .63(.12)  *** | 1.01(.15)  *** | .64(.13)  *** |
| **Random Part** | | | | | | | |
| Between-level $\sqrt{\psi}$ | .117 | .148 | .134 | .134 | .136 | .153 | .157 |
| Within-level $\sqrt{\theta}$ | .800 | .779 | .779 | .796 | .795 | .774 | .791 |
| **Model comparison** | | | | | | | |
| Log-likelihood | -490.3 | -478.4 | -479.6 | -486.7 | -449.8 | -439.9 | -447.5 |
| R^2^ | N/A | .038 | .043 | .002 | .005 | .046 | .005 |

Coefficient (Standard Error)

* p<0.05; ** p<0.01; *** p<0.001
