# Supplementary material for: Microbiological Evaluation of Household Drinking Water Treatment in Rural China Shows Benefits of Electric Kettles: A Cross-Sectional Study
Source: PLoS One. 2015 Sep 30;10(9):e0138451. doi: 10.1371/journal.pone.0138451 (PMC4589372; doi:10.1371/journal.pone.0138451)
Supplement: S2 Table — (DOCX) [file pone.0138451.s006.docx]

Table S2. Log_10_TTC coefficients for Models 7-10.

|  | ***Model Number*** | | | | | |
| --- | --- | --- | --- | --- | --- | --- |
|  | ***7*** | ***8.1*** | ***8.2*** | ***9.1*** | ***9.2*** | ***10*** |
| **Fixed Part** | | | | | | |
| Boil electric kettle [vs. no] | -.57(.13)  *** | -.61(.13)  *** | -.61(.13)  *** | -.62(.13)  *** | -.62(.13)  *** | -.60(.13)  *** |
| Boil pot [vs. no] | -.37(.14)  ** | -.45(.14)  ** | -.45(.14)  ** | -.44(.14)  ** | -.46(.14)  ** | -.44(.14)  ** |
| Drink bottled water [vs. no] | -.43(.12)  *** | -.44(.12)  *** | -.44(.12)  *** | -.45(.13)  *** | -.46(.13)  *** | -.45(.13)  *** |
| Improved water source [vs. no] | -.07(.09) | -.04(.09) | -.04(.09) | -.05(.10) | -.05(.10) | -.04(.10) |
| Safe water storage [vs. no] | -.05(.12) | -.08(.12) | -.08(.12) | -.08(.12) | -.07(.12) | -.05(.12) |
| HH head is literate [vs. no] |  | -.21(.09)  * | -.21(.09)  * | -.20(.10)  * | -.19(.10)  * | -.17(.10) |
| HH head’s age [10 year steps] |  | .03(.03) | .03(.03) | .04(.03) | .03(.03) | .04(.03) |
| HH population |  | -.00(.02) |  |  |  |  |
| TVs by HH population |  | -.38(.20) | -.36(.19) | -.36(.19) | -.35(.19) | -.34(.19) |
| Minutes to health clinic [10  minute steps] |  |  |  | -.03(.05) |  |  |
| Bottled water price/village |  |  |  | .58(.70) | .56(.70) | .72(.72) |
| Improved latrine [vs. no] |  |  |  | -.03(.11) |  |  |
| Wash post defecation [vs. no] |  |  |  |  |  | .07(.09) |
| Soap likely used [vs. no] |  |  |  |  |  | -.06(.09) |
| Wash before meals [vs. no] |  |  |  |  |  | -.20(.13) |
| Intercept | 1.01(.16)  *** | 1.15(.27)  *** | 1.14(.26)  *** | .91(.39)  * | .91(.39)  * | .92(.40)  * |
| **Random Part** | | | | | | |
| Between-level $\sqrt{\psi}$ | .172 | .162 | .162 | .167 | .166 | .167 |
| Within-level $\sqrt{\theta}$ | .771 | .758 | .757 | .760 | .757 | .758 |
| **Model comparison** | | | | | | |
| Log-likelihood | -437.8 | -432.4 | -429.4 | -426.5 | -428.5 | -428.1 |
| R^2^ | .045 | .083 | .083 | .081 | .081 | .081 |

HH=household

Coefficient (Standard Error)

* p<0.05; ** p<0.01; *** p<0.001
