# Supplementary material for: Microbiological Evaluation of Household Drinking Water Treatment in Rural China Shows Benefits of Electric Kettles: A Cross-Sectional Study
Source: PLoS One. 2015 Sep 30;10(9):e0138451. doi: 10.1371/journal.pone.0138451 (PMC4589372; doi:10.1371/journal.pone.0138451)
Supplement: S3 Table — (DOCX) [file pone.0138451.s007.docx]

Table S3. Sample and government data comparisons.

| **County** | **Mean number of adults per HH** | | | **Mean number of children per HH** | | | **Percentage male-headed HHs** | | | **Mean head of HH age** | | |
| --- | --- | --- | --- | --- | --- | --- | --- | --- | --- | --- | --- | --- |
|  | **C** | **S** | **D** | **C** | **S** | **D** | **C** | **S** | **D** | **C** | **S** | **D** |
| A | 3.8 | 3.5 | 0.2 | 1.0 | 1.1 | -0.1 | 82.0 | 71.7 | 10.3 | 42.3 | 51.0 | -8.8 |
| B | 3.4 | 3.6 | -0.2 | 0.8 | 1.1 | -0.3 | 83.9 | 96.2 | -12.3 | 57.6 | 54.0 | 3.7 |
| Overall mean | 3.6 | 3.6 | 0.0 | 0.9 | 1.1 | -0.2 | 82.9 | 83.1 | -0.3 | 49.4 | 52.4 | -3.0 |
| Overall SD | 0.6 | 0.5 | 0.7 | 0.3 | 0.2 | 0.4 | 12.8 | 16.0 | 20.6 | 11.1 | 3.1 | 11.6 |

C=CCDC data | S=Survey data | D=Difference (CCDC-survey) | HH=household | SD=Standard deviation
