# Supplementary material for: Microbiological Evaluation of Household Drinking Water Treatment in Rural China Shows Benefits of Electric Kettles: A Cross-Sectional Study
Source: PLoS One. 2015 Sep 30;10(9):e0138451. doi: 10.1371/journal.pone.0138451 (PMC4589372; doi:10.1371/journal.pone.0138451)
Supplement: S4 Table — (DOCX) [file pone.0138451.s008.docx]

Table S4. Primary HWT method proportions: Sample and population estimates.

|  | **Sample** | | **Population estimates  (using sample weights)** | |
| --- | --- | --- | --- | --- |
|  | **TTC outliers removed** | **All data** | **TTC outliers removed** | **All data** |
| **HWT overview** | | | | |
| Boil (all) | .479 (.430-.528) | .481 (.434-.572) | .476 (.383-.569) | .475 (.377-.572) |
| Purchase bottled water | .345 (.298-.392) | .351 (.307-.396) | .351 (.228-.472) | .344 (.222-.465) |
| Drink untreated water | .176 (.134-214) | .168 (.133-.203) | .174 (.106-.241) | .182 (.113-.250) |
| Total | 1 | 1 | 1 | 1 |
| **HWT overview with boiling disaggregated** | | | | |
| Boil: Electric kettles | .270 (.227-.314) | .273 (.231-.314) | .273 (.182-.364) | .271 (.172-.370) |
| Boil: Pots | .208 (.169-.248) | .208 (.170-.246) | .203 (.117-.289) | .203 (.115-.291) |
| Purchase bottled water | .345 (.298-.392) | .351 (.307-.396) | .351 (.228-.472) | .344 (.222-.465) |
| Drink untreated water | .176 (.134-214) | .168 (.133-.203) | .174 (.106-.241) | .182 (.113-.250) |
| Total | 1 | 1 | 1 | 1 |
