# Supplementary material for: Microbiological Evaluation of Household Drinking Water Treatment in Rural China Shows Benefits of Electric Kettles: A Cross-Sectional Study
Source: PLoS One. 2015 Sep 30;10(9):e0138451. doi: 10.1371/journal.pone.0138451 (PMC4589372; doi:10.1371/journal.pone.0138451)
Supplement: S5 Table — (DOCX) [file pone.0138451.s009.docx]

Table S5. Sensitivity analysis: HWT coefficients part I.

|  | **OLS** | **MLE** | **MLE sample weights level:** | | | **Soap present** | **Very safe storage** | **Less strict PD wash** |
| --- | --- | --- | --- | --- | --- | --- | --- | --- |
|  |  |  | **1** | **2** | **1 & 2** |  |  |  |
| Electric kettle | -.61(.13)  *** | -.60(.13)  *** | -.59(.14)  *** | -.59(.14)  *** | -.59(.14)  *** | -.59(.13)  *** | -.61(.13)  *** | -.59(.13)  *** |
| Pot | -.46(.14)  ** | -.44(.14)  ** | -.44(.15)  ** | -.44(.15)  ** | -.44(.15)  *** | -.44(.14)  ** | -.46(.14)  ** | -.44(.14)  ** |
| Bottled water | -.45(.12)  *** | -.45(.12)  *** | -.44(.16)  ** | -.44(.16)  ** | -.44(.17)  * | -.44(.13)  *** | -.44(.13)  ** | -.44(.13)  *** |

Coefficient (Standard Error)

* p<0.05; ** p<0.01; *** p<0.001
