# Supplementary material for: Microbiological Evaluation of Household Drinking Water Treatment in Rural China Shows Benefits of Electric Kettles: A Cross-Sectional Study
Source: PLoS One. 2015 Sep 30;10(9):e0138451. doi: 10.1371/journal.pone.0138451 (PMC4589372; doi:10.1371/journal.pone.0138451)
Supplement: S6 Table — (DOCX) [file pone.0138451.s010.docx]

Table S6. Sensitivity analysis: HWT coefficients part II.

|  | **Without data collected by enumerator # (n):** | | | | | **w/o storage** | **w/o source** | **w/o storage & source** |
| --- | --- | --- | --- | --- | --- | --- | --- | --- |
|  | **1 (34)** | **10 (22)** | **16 (27)** | **18 (31)** | **22 (27)** |  |  |  |
| Electric kettle | -.58(.13)  *** | -.60(.13)  *** | -.51(.13)  *** | -.60(.14)  *** | -.62(.13)  *** | -.59(.12)  *** | -.60(.13)  *** | -.6(.12)  *** |
| Pot | -.47(.15)  ** | -.40(.15)  ** | -.34(.14)  * | -.43(.15)  ** | -.5 (.14)  *** | -.45(.14)  ** | -.43(.14)  ** | -.45(.13)  ** |
| Bottled water | -.47(.13)  *** | -.46(.13)  *** | -.42(.13)  ** | -.42(.13)  ** | -.49(.13)  *** | -.5(.12)  *** | -.44(.12)  *** | -.48(.12)  *** |

Coefficient (Standard Error)

* p<0.05; ** p<0.01; *** p<0.001
