# Supplementary material for: Microbiological Evaluation of Household Drinking Water Treatment in Rural China Shows Benefits of Electric Kettles: A Cross-Sectional Study
Source: PLoS One. 2015 Sep 30;10(9):e0138451. doi: 10.1371/journal.pone.0138451 (PMC4589372; doi:10.1371/journal.pone.0138451)
Supplement: S7 Table — (DOCX) [file pone.0138451.s011.docx]

Table S7. Sensitivity analysis: HWT coefficients part III.

|  | **Without hand washing:** | | | **MLM w/3 levels** | **With “random” dependent variable:** | | | | |
| --- | --- | --- | --- | --- | --- | --- | --- | --- | --- |
|  | **BM** | **PD** | **BM & PD** |  | **Respondent age** | **Survey duration** | | **Enumerator id.** | **HH id. code** |
| Electric kettle | -.61(.13)  *** | -.60(.13)  *** | -.61(.13)  *** | -.59(.13)  *** | -2.45 (2.10) | | -.6 (1.14) | .4 (.55) | .53 (1.41) |
| Pot | -.44(.14)  ** | -.44(.14)  ** | -.44(.14)  ** | -.45(.14)  ** | -.04 (2.28) | | -.67 (1.23) | -.54 (.61) | 1.16 (1.58) |
| Bottled water | -.45(.13)  *** | -.44(.13)  *** | -.45(.13)  *** | -.45(.13)  *** | 1.51 (2.04) | | .19 (1.1) | .59 (.56) | 1.1 (1.45) |

Coefficient (Standard Error) | HH=household

* p<0.05; ** p<0.01; *** p<0.001
