# Supplementary material for: Microbiological Evaluation of Household Drinking Water Treatment in Rural China Shows Benefits of Electric Kettles: A Cross-Sectional Study
Source: PLoS One. 2015 Sep 30;10(9):e0138451. doi: 10.1371/journal.pone.0138451 (PMC4589372; doi:10.1371/journal.pone.0138451)
Supplement: S8 Table — (DOCX) [file pone.0138451.s012.docx]

Table S8. Sensitivity analysis: Null Model and Model 10 with TTC outliers included.

|  | ***Null Model*** | ***Model 10*** |
| --- | --- | --- |
| **Fixed Part** | | |
| Boil with electric kettle [vs. no] |  | -.68(.12)*** |
| Boil with pot [vs. no] |  | -.49(.13)*** |
| Drink bottled water [vs. no] |  | -.56(.12)*** |
| Improved water source [vs. no] |  | -.08(.09) |
| Safe water storage [vs. no] |  | -.06(.12) |
| HH head is literate [vs. no] |  | -.11(.09) |
| HH head’s age [10 year steps] |  | .02(.03) |
| TVs by HH population |  | -.32(.18) |
| Bottled water price by village |  | .62(.65) |
| Wash post defecation [vs. no] |  | .04(.09) |
| Soap likely used [vs. no] |  | -.07(.08) |
| Wash before meals [vs. no] |  | -.21(12) |
| Intercept | .55(.04)*** | 1.13(.37)** |
| **Random Part** | | |
| Between-level $\sqrt{\psi}$ | .091 | .141 |
| Within-level $\sqrt{\theta}$ | .800 | .756 |
| **Model comparison** | | |
| Log-likelihood | -535.2 | -468.2 |
| R^2^ | N/A | .089^a^ |

HH=household

Coefficient (Standard Error)

* p<0.05; ** p<0.01; *** p<0.001

^a^ R^2^ calculated based on total variance from the Null Model with 38 TTC outliers included
