# Supplementary material for: Microbiological Evaluation of Household Drinking Water Treatment in Rural China Shows Benefits of Electric Kettles: A Cross-Sectional Study
Source: PLoS One. 2015 Sep 30;10(9):e0138451. doi: 10.1371/journal.pone.0138451 (PMC4589372; doi:10.1371/journal.pone.0138451)
Supplement: S9 Table — (DOCX) [file pone.0138451.s013.docx]

Table S9. Risk ratios for TTC by HWT method: Electric kettles as reference (and outliers).

|  | **38 TTC outliers removed** | | **All data (TTC outliers included)** | |
| --- | --- | --- | --- | --- |
|  | **Risk Ratio (95% CI)** | **p-value** | **Risk Ratio (95% CI)** | **p-value** |
| Boil: Electric kettles | 1* | n/a | 1* | n/a |
| Boil: Pots | 1.51 (1.02-2.22) | 0.0370 | 1.55 (1.06-2.27) | 0.0247 |
| Bottled water | 1.42 (0.99-2.03) | 0.0523 | 1.36 (0.95-1.96) | 0.0881 |
| Untreated water | 2.03 (1.42-2.90) | 0.0001 | 2.25 (1.59-3.19) | 0.0000 |

*Reference for unadjusted risk ratios (no TTC detected = 0)
