# Supplementary material for: Microbiological Evaluation of Household Drinking Water Treatment in Rural China Shows Benefits of Electric Kettles: A Cross-Sectional Study
Source: PLoS One. 2015 Sep 30;10(9):e0138451. doi: 10.1371/journal.pone.0138451 (PMC4589372; doi:10.1371/journal.pone.0138451)
Supplement: S10 Table — (DOCX) [file pone.0138451.s014.docx]

Table S10. Risk ratios for diarrhea by HWT method: Electric kettles as reference.

|  | **Risk Ratio (95% CI)** | **p-value** |
| --- | --- | --- |
| Boil: Electric kettle | 1* | n/a |
| Boil: Open-pot | 0.66 (0.12-3.50) | 0.6188 |
| Bottled water | 1.39 (0.42-4.63) | 0.5931 |
| Untreated water | 1.63 (0.42-6.31) | 0.4781 |

*Reference for unadjusted risk ratios (no diarrhea reported = 0)
