# Supplementary material for: Microbiological Evaluation of Household Drinking Water Treatment in Rural China Shows Benefits of Electric Kettles: A Cross-Sectional Study
Source: PLoS One. 2015 Sep 30;10(9):e0138451. doi: 10.1371/journal.pone.0138451 (PMC4589372; doi:10.1371/journal.pone.0138451)
Supplement: S11 Table — (DOCX) [file pone.0138451.s015.docx]

Table S11. Comparison of County A replacement and randomly selected villages.

| **Village code** | **Adults per HH:  Mean (SD)** | **Children per HH:  Mean (SD)** | **Male-headed HHs:  %** | **Head of HH age:  Mean (SD)** | **TVs per HH population: Mean (SD)** |
| --- | --- | --- | --- | --- | --- |
| 2, 3, & 8 (replacements) | 3.52 (1.47) | 1.10 (1.16) | 65.17 | 54.36 (13.42) | 0.29 (0.19) |
| 1, 4, 5, 6, & 7 | 3.57 (1.60) | 1.07 (1.02) | 75.84 | 49.02 (12.19) | 0.30 (0.18) |

HH = household | SD = standard deviation
