# Supplementary material for: Microbiological Evaluation of Household Drinking Water Treatment in Rural China Shows Benefits of Electric Kettles: A Cross-Sectional Study
Source: PLoS One. 2015 Sep 30;10(9):e0138451. doi: 10.1371/journal.pone.0138451 (PMC4589372; doi:10.1371/journal.pone.0138451)
Supplement: S12 Table — (DOCX) [file pone.0138451.s016.docx]

Table S12. Total Coliform summary statistics by HWT method.

|  | **Minimum** | **Median** | **Maximum** | **Mean** | **Standard Deviation** | **n** |
| --- | --- | --- | --- | --- | --- | --- |
| **Total Coliforms (MPN/100mL): Raw data*** | | | | | | |
| Electric kettles | 0 | 215 | 24,000 | 2,027.183 | 3,842.871 | 120 |
| Pots | 0 | 475 | 23,000 | 3,451.293 | 5,578.817 | 92 |
| Bottled water | 0 | 680 | 18,000 | 2,481.265 | 3,887.922 | 155 |
| Untreated | 0 | 420 | 8,800 | 1,369.293 | 1,992.42 | 75 |
| **Total Coliforms (MPN/100mL): Raw data with outliers removed*** | | | | | | |
| Electric kettles | 0 | 97 | 24,000 | 1,642.495 | 3,544.952 | 109 |
| Pots | 0 | 445 | 23,000 | 3,290.024 | 5,606.744 | 84 |
| Bottled water | 0 | 465 | 18,000 | 1,992.114 | 3,506.331 | 140 |
| Untreated | 0 | 540 | 8,800 | 1,442.493 | 2,023.49 | 71 |
| **Total Coliforms (MPN/100mL): Log_10_ transformed**** | | | | | | |
| Electric kettles | 0 | 2.311625 | 4.380211 | 2.263636 | 1.27177 | 120 |
| Pots | 0 | 2.676477 | 4.361728 | 2.567493 | 1.243417 | 92 |
| Bottled water | 0 | 2.832509 | 4.255272 | 2.696099 | 1.025783 | 155 |
| Untreated | 0 | 2.623249 | 3.944483 | 2.352868 | 1.13787 | 75 |
| **Total Coliforms (MPN/100mL): Log_10_ transformed with outliers removed**** | | | | | | |
| Electric kettles | 0 | 1.986772 | 4.380211 | 2.130249 | 1.248553 | 109 |
| Pots | 0 | 2.648333 | 4.361728 | 2.514629 | 1.245468 | 84 |
| Bottled water | 0 | 2.667428 | 4.255272 | 2.580656 | 1.009998 | 140 |
| Untreated | 0 | 2.732394 | 3.944483 | 2.388775 | 1.156631 | 71 |

HWT = Household water treatment

*Values below the detection limit were converted to zero

**Values below the detection limit were converted to one before the log transformation

**Notes:** Samples from electric kettles had significantly lower mean TC contamination (Log_10_TC=2.13 MPN/100mL) than samples from bottled water (Log_10_TC=2.58 MPN/100mL) (Scheffe’s multiple-comparison test, p=0.026; Bonferroni test, p=0.014). However, this was the only statistically significant association of note relative to HWT methods. There was no association between TC concentrations and improved or unimproved water sources.
